# Urinary charged metabolite profiling of colorectal cancer using capillary electrophoresis-mass spectrometry

**DOI:** 10.1038/s41598-020-78038-2

**Published:** 2020-12-03

**Authors:** Ryutaro Udo, Kenji Katsumata, Hiroshi Kuwabara, Masanobu Enomoto, Tetsuo Ishizaki, Makoto Sunamura, Yuichi Nagakawa, Ryoko Soya, Masahiro Sugimoto, Akihiko Tsuchida

**Affiliations:** 1 Department of Gastrointestinal and Pediatric Surgery, Tokyo Medical University, 6-7-1, Nishijinjuku, Shinjuku, Tokyo, 160-0023 Japan; 2 Institute for Advanced Biosciences, Keio University, Tsuruoka, Yamagata 997-0811 Japan; 3 Research and Development Center for Minimally Invasive Therapies, Medical Research Institute, Tokyo Medical University, 6-1-1, Shinjuku, Tokyo, 160-0022 Japan; 4 Department of Gastroenterological Surgery and Transplantation Surgery, Tokyo Medical University Hachioji Medical Center, 1163 Tatemachi, Hachioji City, Tokyo 193-0998 Japan

**Keywords:** Biomarkers, Diagnostic markers

## Abstract

Colorectal cancer (CRC) has increasing global prevalence and poor prognostic outcomes, and the development of low- or less invasive screening tests is urgently required. Urine is an ideal biofluid that can be collected non-invasively and contains various metabolite biomarkers. To understand the metabolomic profiles of different stages of CRC, we conducted metabolomic profiling of urinary samples. Capillary electrophoresis-time-of-flight mass spectrometry was used to quantify hydrophilic metabolites in 247 subjects with stage 0 to IV CRC or polyps, and healthy controls. The 154 identified and quantified metabolites included metabolites of glycolysis, TCA cycle, amino acids, urea cycle, and polyamine pathways. The concentrations of these metabolites gradually increased with the stage, and samples of CRC stage IV especially showed a large difference compared to other stages. Polyps and CRC also showed different concentration patterns. We also assessed the differentiation ability of these metabolites. A multiple logistic regression model using three metabolites was developed with a randomly designated training dataset and validated using the remaining data to differentiate CRC and polys from healthy controls based on a panel of urinary metabolites. These data highlight the changes in metabolites from early to late stage of CRC and also the differences between CRC and polyps.

## Introduction

More than 1.8 million new cases of colorectal cancer (CRC) and 881,000 deaths are estimated to have occurred in 2018. CRC ranks third in terms of incidence and second in terms of mortality among cancers worldwide^1^. The incidence and mortality of CRC show very similar patterns in both men and women, despite showing large geographical variation. The detection and removal of cancer precursors can reduce CRC incidence and mortality. Therefore, the early detection of CRC is needed to enable less invasive treatments and results in lower morbidity and mortality.

Serum carcinoembryonic antigen (CEA) and carbohydrate antigen 19-9 (CA19-9) have been used as biomarkers for CRC. However, serum CEA is elevated in only approximately 50% of patients with CRC who have lymph node metastasis, and in 75% of patients with distant metastasis. Serum CA19-9 is positive in more than 70% of patients with distant metastasis and is useful for the diagnosis of recurrence and metastasis of CRC^2^. The sensitivity of serum CEA and CA19-9 is extremely low for the detection of stage II CRCs, at 33% and 11%, respectively^3–6^. Therefore, more accurate screening methods and novel biomarkers for the detection of CRC are required.

A less invasive screening test has been widely used in Japan for the detection of CRC, which detects microscopic amounts of blood by targeting either heme or human globin in the stool^7^. However, the rate of early detection of CRC remains only 37%^8–11^. This is because many patients refuse to undergo colonoscopy because of stigma, difficulty in preparing for the test, and the physical burden of the test. Some patients may not undergo an endoscopic examination because the rate of a positive diagnosis even after a positive faecal occult blood test is low. Furthermore, while people in Japan have access to high-quality medical care through the universal health insurance system, this may reduce patients’ awareness of the importance of preventive medicine. Therefore, the development of less invasive screening tools for early detection of CRC, even in non-symptomatic patients, should be a priority.

There are many reports describing dynamic reprogramming of metabolic pathways in CRC^12,13^. The proto-oncogene *MYC* regulates various metabolic pathways in CRC, including the glycolysis^14^ and tryptophan pathways^15^. *MYC* also activates polyamine synthesis in CRC by activating the first rate-limiting enzyme, ornithine decarboxylase^16,17^. Spermine/spermidine *N*^1^-acetyltransferase, *N*^1^-acetylpolyamine oxidase, and spermine oxidase are concurrently activated to metabolize spermine to *N*^1^-acetylspermine, spermidine to *N*^1^-acetylspermidine, and spermine to spermidine, respectively, and these metabolites are secreted into the surrounding tissues^18–20^. In particular, an increase in urinary *N*^1^,*N*^12^-diacetylspermine has been reported in CRC patients^21–23^. However, it is difficult to specifically diagnose CRC using this single marker on its own, because an increase in this metabolite in the blood of lung cancer patients and in the urine of breast cancer patients has also been reported. We previously performed a targeted analysis of urinary polyamines using liquid chromatography-triple quadrupole mass spectrometry (MS) and confirmed the elevation of *N*^1^,*N*^12^-diacetylspermine in urine samples from patients with CRC^9^.

Metabolomics has been used for the detection of metabolic abnormalities caused by tumours in biofluid samples that can be collected by minimally invasive methods, such as blood^24,25^ and urine^26,27^. Potential biomarkers for the detection of CRC include metabolites that are dysregulated during the progression of CRC^28^. The combination of multiple markers into a biomarker panel has the potential to differentiate CRC from polyps^27,29^. Therefore, comprehensive metabolomic analysis using a sophisticated classification method is expected to contribute to the identification of patients with CRC.

In the present study, we utilized capillary electrophoresis-mass spectrometry (CE-MS) to perform non-targeted analysis of urine collected from patients with CRC or polyps (P), as well as from healthy controls, to profile hydrophilic urinary metabolites. To understand the change in metabolomic patterns depending on the progression, we conducted pathway analyses of the profiled metabolites. The discriminating ability of these profiles was assessed using a multiple logistic regression model (MLR).

## Materials and methods

### Study subjects

This study was conducted according to the guidelines of the Declaration of Helsinki. The study protocol was approved by the Ethics Committee of Tokyo Medical University (study approval no. 2346). Written informed consent was obtained from each subject before participation in the study. Patients with CRC included subjects who underwent chemotherapy. Patients with chronic metabolic diseases, such as diabetes, were included.

The patients’ clinical data were obtained as described in a previous study^9^. Briefly, urinary samples were collected from patients with CRC (n = 209) and P (n = 16). Urinary samples were also collected from healthy controls (n = 22). Among them, five subjects provided multiple samples at various time (7:00–8:00, 11:00–12:00, and 17:00–18:00) on three consecutive days. Six to nine samples were collected from identical persons because of several missing samples. The first collected samples were defined as healthy control 1 (HC1, n = 22) (these samples were collected at 9:00 on the first day) and the other samples were defined as healthy control 2 (HC2, n = 50). The samples from patients with P and CRC were collected once between 9:00–16:00.

The resected specimens were pathologically classified according to the seventh edition of the Union for International Cancer Control TNM classification of malignant tumours. Serum CEA and CA19-9 levels were measured using a radioimmunoassay (Abbott, Chiba, Japan). The limit of detection of CEA was 0.5 ng/mL and that of CA19-9 was 2 U/mL. A high CEA level was defined as a level exceeding 5 ng/mL, and a high CA19-9 level was defined as a level exceeding 37 U/mL, according to the guidelines defined by the manufacturer of the test kit.

### Metabolomics of urine samples

Urine was collected as previously described^9^. The collected samples were divided into two tubes and stored at − 80 °C. One of the samples was used for liquid chromatography-mass spectrometry (LC–MS)-based polyamine analysis in our previous study^9^ and the other was used in the present study for CE-MS-based metabolomic profiling. Frozen urine samples were thawed at 4 °C for approximately 1.5 h and subsequently homogenised using a vortex mixer at room temperature, and centrifuged through a 5-kDa cutoff filter (Millipore, Bedford, MA) at 9100 × *g* for at least 2.5 h at 4 °C. Twenty microlitre of each sample was placed in a 1.5 mL Eppendorf tube with 80 μL of Milli-Q water including 2 mM each of methionine sulfone, 2-[N-morpholino]-ethanesulfonic acid, D-camphor-10-sulfonic acid (sodium salt), 3-aminopyrrolidine, and trimesate.

The mixture was centrifugally filtered at 9100 × *g* for 30 min at 4 °C through a 5-kDa cutoff filter (Millipore) to remove large molecules. The filtrate was transferred to a vial and assayed by CE-time-of-flight (TOF)-MS. The parameters of CE-TOF-MS have been described elsewhere and were applied with slight modifications, as follows^30^. Each sample was measured in both positive and negative mode. The parameters in the negative mode were the same as those described previously^30^. For the measurement of cations, two conditions were modified as follows: (1) fused silica capillaries (50 µm i.d. × 95 cm total length) were used, and (2) before each injection, the capillary was equilibrated for 4 min by flushing with 1 M formate. To quantify each metabolite, standard mixtures, including 20 μL of each metabolite and 200 μL of each standard compound, were measured before analysis of the samples.

Raw CE-TOF–MS data were analysed with our proprietary software, MasterHands (ver. 2.17.2.15, Keio University, Yamagata, Japan)^31,32^. The raw data were adjusted by 0.02 m*/z* each to generate electropherograms. For each electropherogram, the background noise was removed and possible peaks with a signal-to-noise ratio of greater than 2.0 were retained. Peaks that were detected in multiple samples were aligned, missing peaks were filled, and redundant features, for example, isotopic peaks, fragments, and noise peaks were eliminated. Metabolite identities were assigned to each feature by matching the corresponding *m/z* values and normalized migration times of standard compounds. The absolute concentration of each metabolite peak was calculated by dividing the peak area of by that of the internal standards (methionine sulfone and D-camphor-10-sulfonic acid (sodium salt) for cations and anions, respectively), and eliminating the drift in MS sensitivity. These absolute concentrations were normalised to the creatinine concentration of each sample and used for subsequent analysis.

### Data analysis

To compare the metabolomic profiles of HC1, P, and CRC, we performed clustering analyses, principal component analysis (PCA), and partial least squares-discrimination analyses (PLS-DA). PLS-DA was also conducted by adding HC2 data, for evaluating the variation in data for healthy controls. To evaluate the discrimination ability of the combination of metabolites, two commonly used feature selection methods were employed. First, support vector machine-feature selection (SVM-FS) was initially performed to rank the identified metabolites by differential ability based on the margin of the data between HC1 and CRC^33^. Second, among the highly ranked metabolites, correlation-based feature selection (CFS) was performed to select a minimum set of features^34^. Stepwise feature selection (backward method) was also performed to eliminate unnecessary features. Features with a *P*-value greater than 0.05 were eliminated from the model. The MLR model was developed using highly ranked features. To assess the discriminatory ability of these potential biomarkers, we randomly split the data into a training and a validation dataset, while maintaining the ratio of HC1, P, and CRC subjects, with half of the data used as the training data and half used as the validation data. The MLR model was used to identify metabolites that could differentiate between two groups. We developed the MLR model to differentiate CRC + P from HC1, for evaluating the potential for detecting these diseases by non-invasive screening. As an internal validation, 10-fold cross-validation (CV) was performed using the training dataset. As external validation, the developed MLR model was evaluated using the validation data.

To eliminate potential bias caused by the random split, multiple times two-fold CV was performed using all the datasets. The selection of features was not performed in these experiments, and features selected in the previous experiment were used. Because of the limited sample size, resampling tests were also performed, that is, datasets were generated by random selection from the original datasets, which enabled redundant selection, and the MLR model was built and evaluated using the generated datasets.

Mann–Whitney and Kruskal–Wallis tests followed by the Dunn’s post hoc test were used to analyse the differences in metabolite levels among 2 and 3 of the groups, respectively. The χ^2^ test was used to assess gender bias in the dataset. For multiple testing correction, *P*-values were adjusted using a false discovery rate (FDR). The areas under the receiver operating characteristic curves were used to analyse the differentiation ability of the MLR models.

Pathway analyses, PCA, and PLS-DA were conducted using MetaAnalyst^35^. Log-transformation and mean centring options without sample normalization were used for PCA and PLS-DA. The SVM-FS and CFS were performed using the default parameters in Waikato Environment for Knowledge Analysis (Weka) (ver. 3.6.15, New Zealand)^36^. The metabolites ranked within 20% by SVM-FS were used for subsequent feature selection. Both the two-fold CV and resampling tests were performed 200 times with different random values. GraphPad Prism (ver. 8.4.2, GraphPad Software Inc., San Diego, CA, USA), JMP Pro (ver 14.1.0, SAS Institute Inc., Cary, NC, USA), R-language (ver 4.0.2, R foundation for statistical computing, Vienna, Austria), and MeV TM4 (ver. 4.7.4, http://mev.tm4.org/) were used for statistical analysis. The authors will provide raw data upon request.

## Results

### Urinary metabolomic profiling

**Figure 1:**
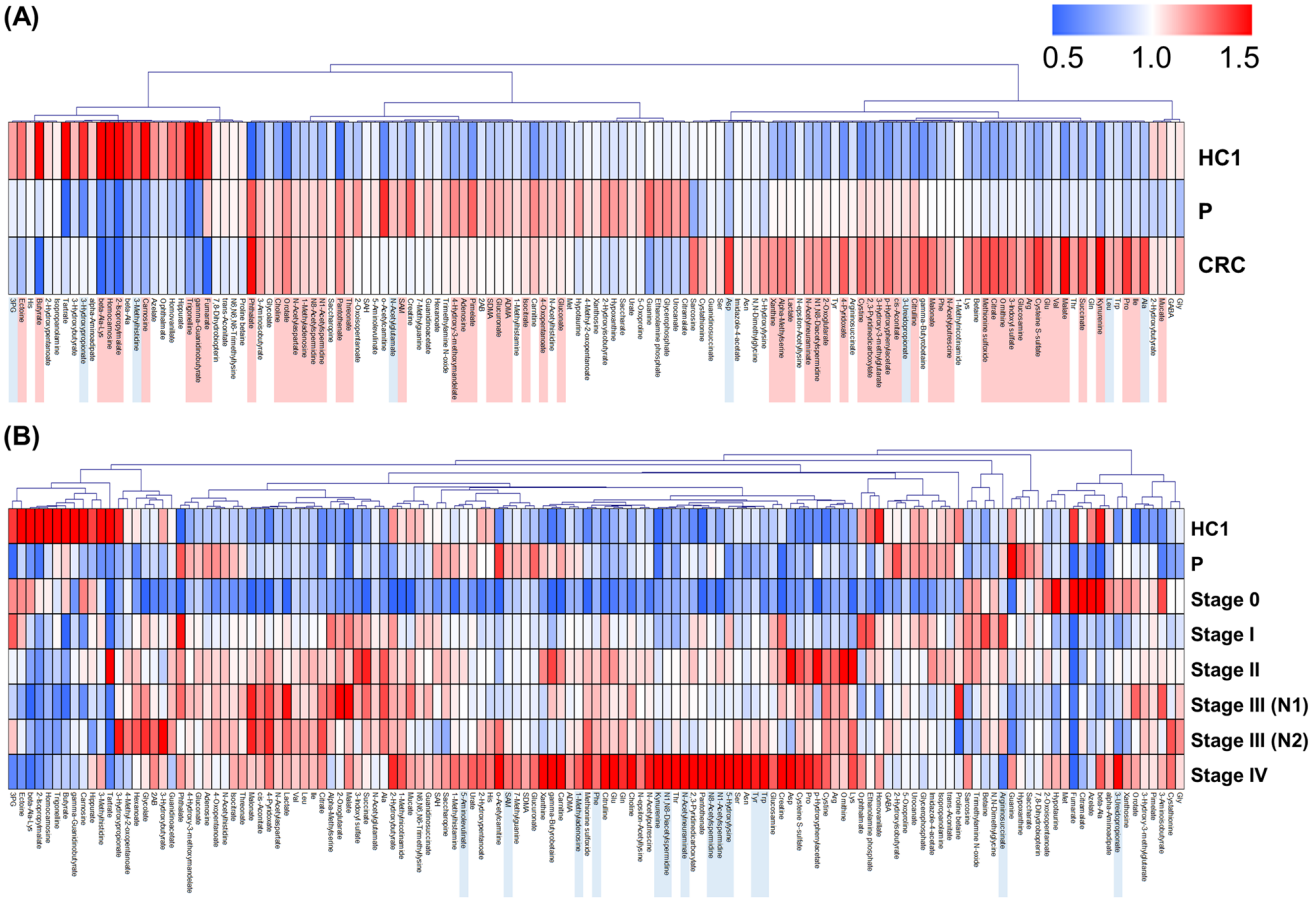
Heatmap of urinary metabolomic profiles. (**A**) Comparison of metabolites identified in the urine of healthy controls 1 (HC1) and patients with polyps (P) and colorectal cancer (CRC). (**B**) Stage-specific profiles of (**A**). Concentrations of each metabolite are represented as the average of all patients per group normalised to the total amount of that metabolite detected. Colours represent higher (red) or lower (blue) concentration compared to the average (white). Pearson’s correlation was used for clustering. Metabolites detected in more than 50% of samples were included in this analysis. Mann–Whitney was conducted between HC1 and CRC for (**A**) and CRC at stage 0 to III and stage IV for (**B**). The metabolites showing *P* < 0.05 are coloured light blue and those showing *P* < 0.05 (adjusted by FDR) are coloured light red. MeV TM4 (ver. 4.7.4, http://mev.tm4.org/) was used for generating heatmaps.

Characteristics of patients in the present cohort are summarized in Tables 1 and 2. Metabolomic analysis successfully identified and quantified 154 metabolites. The metabolites that were frequently identified among the samples were visualized in a heatmap (Fig. 1). HC1, P, and CRC groups clustered according to different metabolite profiles. The HC1 group included the lowest number of metabolites with higher concentrations than in the P and CRC groups. In the comparison between HC1 and CRC, 67 metabolites showed *P* < 0.05 (Mann–Whitney test) and, among them, 59 metabolites showed *P* < 0.05, adjusted by FDR. Histidine, for example, had a higher concentration in the HC1 group than in the other two groups. The P and CRC groups showed substantial overlap, with several metabolites present at higher levels in the P and CRC groups than in the HC1 group; these included choline and single-acetylated polyamines. *N*^1^-acetylspermidine and *N*^1^-acetylspermine levels were higher in the P and CRC groups than in the HC1 group. The levels of methionine were highest in the P group. Most amino acids were present at higher levels in the CRC group (Fig. 1A). The metabolic profiles also showed stage-specific patterns in the CRC group. In particular, the profile of stage IV CRC included several metabolites that were present at a higher concentration compared with other stages (Fig. 1B). The comparison of CRC with stage 0 to III and those with stage IV revealed 14 metabolites with *P* < 0.05 (Mann–Whitney test), whereas no metabolites had *P* < 0.05, adjusted by FDR. The kynurenine and polyamines, such as *N*^1^-acetylspermidine and *N*^8^-acetylspermidine, were included.

**Table 1:** Subject characteristics.

Group	Stage	n	Age		Gender	
			Mean	SD	Male	Female
HC		22	46.8	13.1	17	5
P		16	63.2	11.1	12	4
CRC	0	2	76.5	0.710	2	0
	I	52	68.9	10.6	26	26
	II	67	69.7	11.4	42	25
	III (N1)	46	67.9	14.7	24	22
	III (N2)	25	67.1	11.6	12	13
	IV	17	69.5	9.92	8	9

*CRC* colorectal cancer, *P* polyps, *HC* healthy controls.

**Table 2:** Summarised characteristics of the three clinical groups.

Comparison		Age	Gender
HC	P	0.0001	0.8708
HC	CRC	< 0.0001	0.0407
P	CRC	0.0641	0.1122

Mann–Whitney test and Chi-square test were used for age and gender, respectively.

*CRC* colorectal cancer, *P* polyps, *HC* healthy controls.

**Figure 2:**
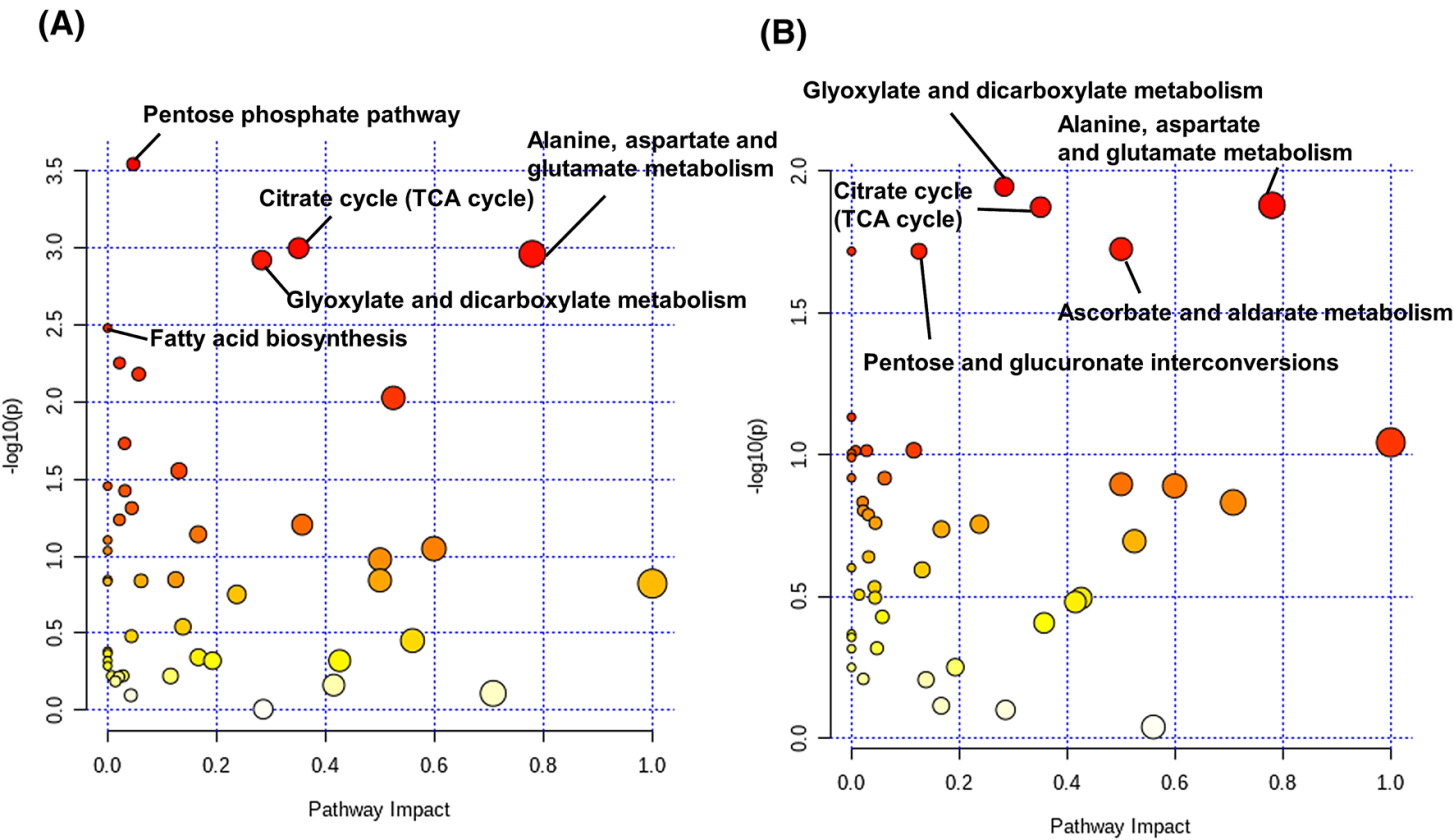
Pathway analyses using MetaboAnalyst (v.4.0, https://www.metaboanalyst.ca). Comparisons of healthy control 1 (HC1) and colorectal cancer (CRC) (**A**) and polyp (P) and CRC groups (**B**). The colour and size of each dots indicate *P*-value and pathway impact values based on the topological analysis, respectively. Yellow and red indicated higher and lower *P*-values. Larger dots indicate a higher impact score. The names of pathways with small *P*-values are described.

We conducted pathway analyses using the profiled metabolite concentrations. The comparison of HC1 and CRC resulted in the highest –log_10_ *P*-value, indicating the lowest *P*-value for the pentose phosphate pathway. Glyoxylate and dicarboxylate metabolism, tricarboxylic acid (TCA) cycle and alanine, aspartate and glutamate metabolism also had high –log_10_ *P*-values (> 2.5) and higher pathway impact values (Fig. 2A). In the comparison of P and CRC, glyoxylate and dicarboxylate metabolism, TCA cycle, and alanine, aspartate and glutamate metabolism, pentose and glucuronate interconversions, ascorbate and aldarate metabolism showed higher –log_10_ *P*-values compared with the other pathways (Fig. 2B). Taken together, the TCA cycle is a CRC-specific difference.

### Differentiation based on urinary metabolites

**Figure 3:**
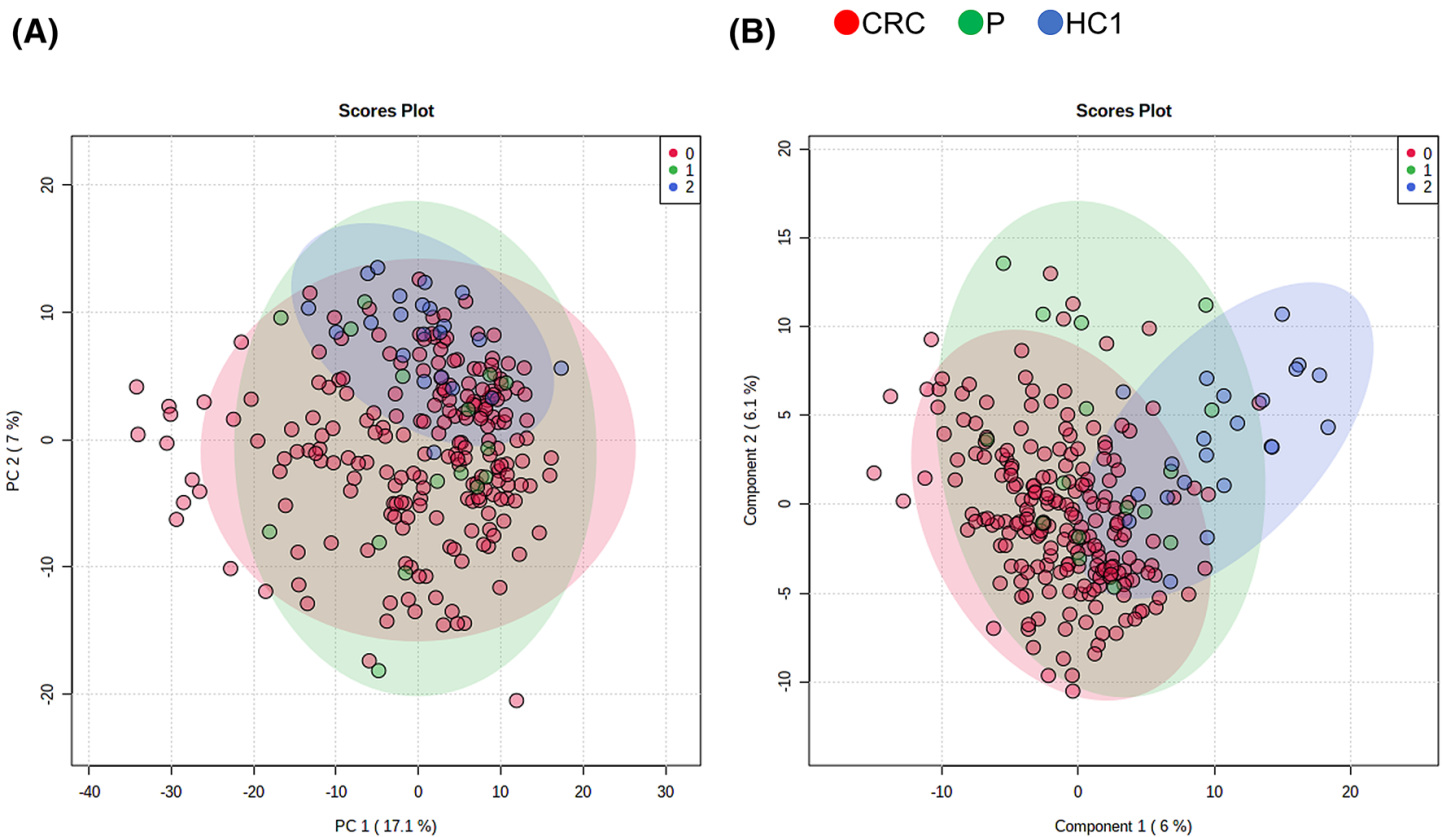
Score plots of principal component analysis (PCA) using MetaboAnalyst (v.4.0, https://www.metaboanalyst.ca). (**A**) and partial least squares-discrimination analyses (PLS-DA) (**B**) of metabolites identified in the urine of healthy control 1 (HC1) and patients with polyps (P) or colorectal cancer (CRC), or healthy controls. The data was log-transformed and mean centred for both the analyses. Loading plots of PCA and the VIP score *R*^2^ and *Q*^2^ are summarized in Figure S1.

We analysed the overall change in the concentrations of the profiled metabolites of the three groups by PCA and PLS-DA (Figs. 3 and S1). The PCA plots showed overlap between all the groups, with the HC1 samples grouped, and the P and CRC samples spread out. The HC1 samples grouped on the right of the PLS-DA, whereas the P and CRC samples were distributed in the area from the centre to the left. The metabolites showing top variable importance in projection (VIP) scores were depicted (Figure S2). Fumarate, 2-isopropylmalate, butyrate, and carnosine showed a significant difference in both HC1 vs P and HC1 vs CRC comparisons (Kruskal–Wallis and Dunn’s post-tests), and the other seven metabolites, such as guanine, showed a significant difference in the HC1 vs CRC comparison.

To assess the variations in the urinary samples collected at different times and days, PLS-DA was conducted using the HC2 data (Figure S3). Most of the plots of P and CRC overlapped at the left side and most of the plots of HC1 and HC2 overlapped at the right side of score plots (Figure S3A). The *Q*^2^ values of the analyses with HC2 (Figure S3C) were higher than those without HC2 (Figure S1C). The metabolites showing high VIP scores, such as fumarate, xanthine, and p-hydroxyphenylacetate, were common in both the PLS-DA analyses (Figure S1B and Figure S3B). These results indicate the high reproducibility bewteen HC1 and HC2 data.

**Figure 4:**
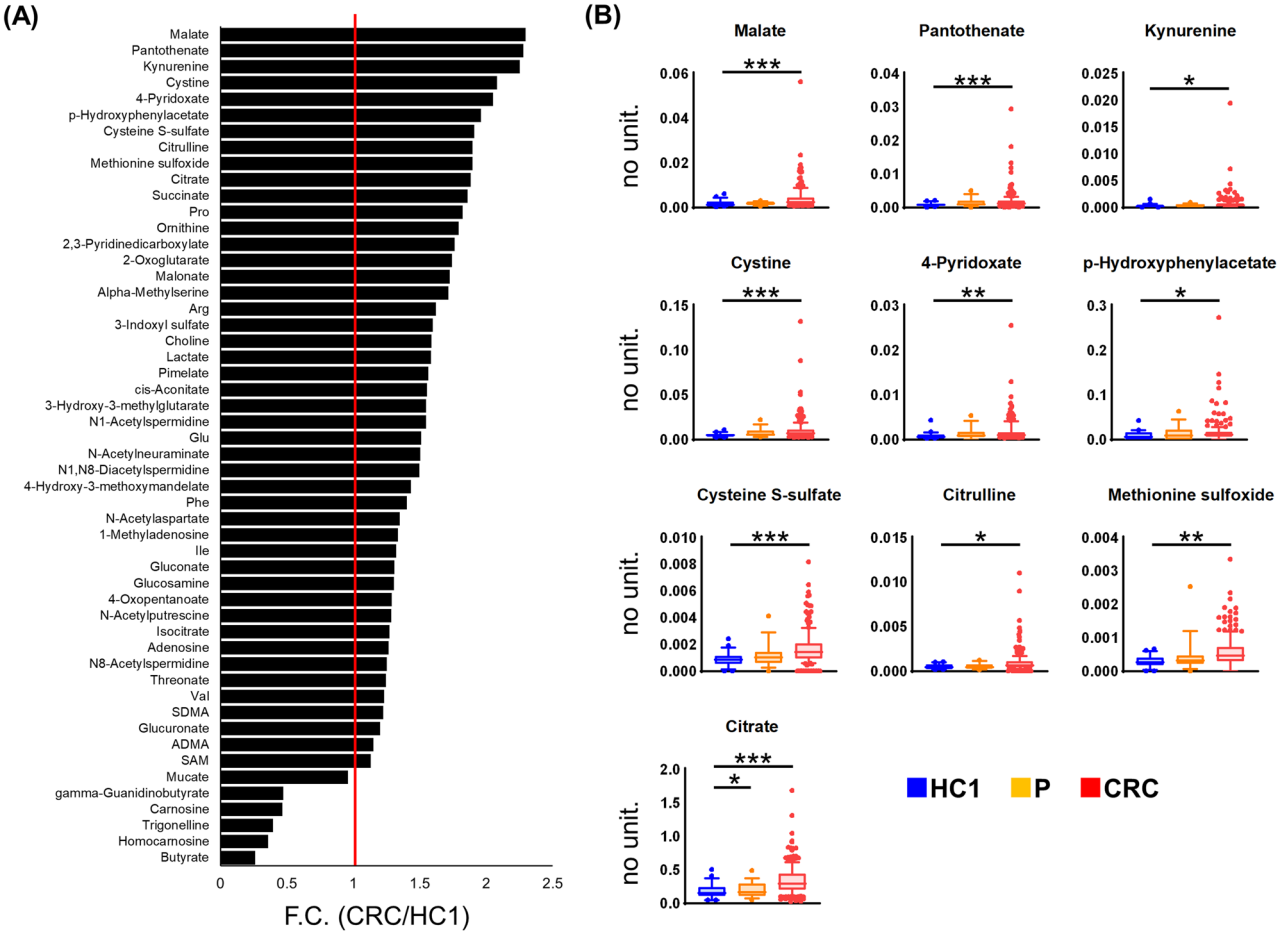
The average fold change of metabolites with significantly different levels between healthy control 1 (HC1) and patients with colorectal cancer (CRC) (Mann–Whitney test; *P* < 0.05 adjusted by FDR) (**A**). The representative box plots of metabolite concentrations within the top tenfold change values (**B**). Metabolite concentrations are normalized to that of creatinine for each sample. The comparison among HC1, polyp (P), and CRC groups was performed using Kruskal–Wallis and Dunn’s post-tests. All metabolites shown here had *P* < 0.005 in the Kruskal–Wallis test, and ***, **, and * indicate *P* < 0.0001, *P* < 0.01, and *P* < 0.05 in the post-tests, respectively. GraphPad Prism (ver. 8.4.2, https://www.graphpad.com/) was used.

The metabolites with statistically significant differences between the CRC and HC1 groups are depicted in Fig. 4. A total of 52 metabolites were present at different levels between these two groups (*P* < 0.05, Mann–Whitney test, adjusted by FDR). The top 10 metabolites with a large fold change (F.C.) difference between these two groups are shown in Fig. 4B and include intermediate metabolites of various pathways, such as malate and citrate (tricarboxylic acid [TCA] cycle), kynurenine (tryptophan pathway), and cystine (pentose phosphate pathway). Among P, CRC, and HC1, only citrate had a statistically significant difference between the HC1 and P groups and between the HC and CRC groups (Kruskal–Wallis test followed by Dunn’s post-hoc test).

**Figure 5:**
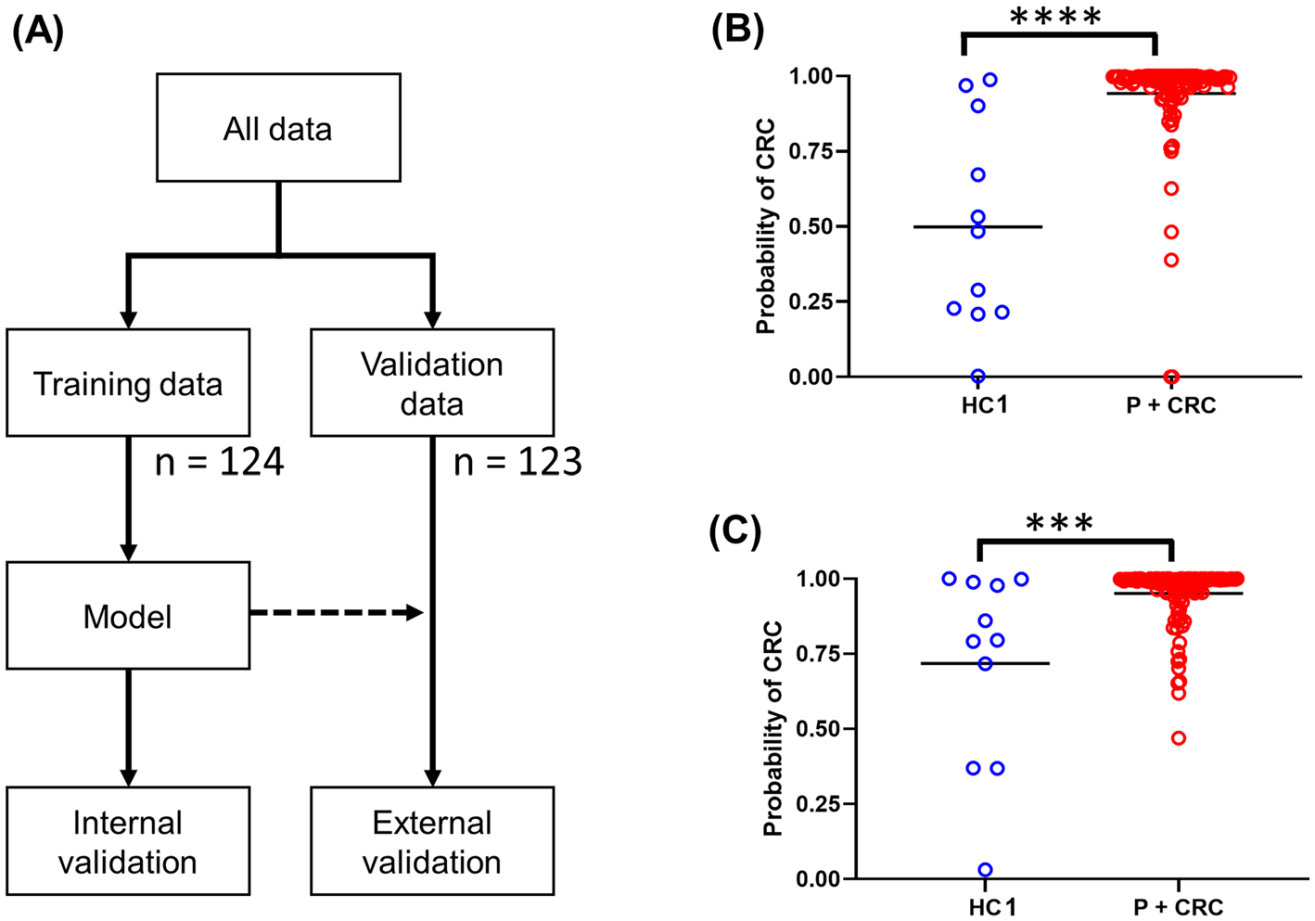
Multiple logistic regression (MLR)-based discrimination tests used for both MLR (**A**) design of data split, (**B**) internal validation and (**C**) external validation results of MLR. The corresponding receiver operating characteristic (ROC) curves are depicted in Figure S2. ****P < 0.0001 and ***P < 0.001. GraphPad Prism (ver. 8.4.2, https://www.graphpad.com/) was used.

We analysed the differentiation ability of a combination of a panel of metabolites. We randomly divided the data, and an MLR model was developed and analysed by tenfold CV using the training data. The model was evaluated using the validation data (Fig. 5A). The MLR differentiated CRC + P from HC1 and the distribution of their predicted probabilities using 10-fold CV and the validation data are shown in Fig. 5B,C. The parameters and selected metabolites of the MLR model are summarised in Table S1. The MLR included butyrate, 3-hydroxy-3-methylglutarate, and carnosine as features. The discriminatory abilities of these putative biomarker panels are summarised in Table S2 and ROC curves are depicted in Figure S4.

Using the selected features, we also developed an MLR model using all the data, that is only numerical coefficients were calculated using the same metabolites (Table S1). This model was also validated using two-fold CV and resampling using all the data (Figure S4C). Based on the ROC curves, the optimal thresholds were calculated for the MLR, and the positive rates based on tumour markers, CEA and CA19-9, predicted by the MLR model, were compared (Table S3). Table S4 summarises the correlation among tumour markers, values predicted by the MLR model, and each metabolite used for the MLR model. Urinary polyamine concentrations in the data from the present study and those reported previously^9^ were compared.

## Discussion

In the present study, non-targeted metabolomic analysis of urine samples collected from subjects with CRC, P, and HC1 was used to understand the changes in the metabolomic profiles. Based on this analysis, the metabolic profiles of each group were unique, and stage-specific differences were observed within the CRC group.

In the CRC group, many metabolites were present at high concentrations, and in particular, the metabolites in the samples of patients with stage IV CRC, which has metastatic potential, were higher than those in the other stages, as well as in the P and HC1 groups (Fig. 1). When comparing HC1 and CRC samples, 52 metabolites were significantly different (FDR-corrected *P* < 0.05) (Fig. 4). However, PCA and PLS-DA analysis were not able to separate these groups based on their metabolic profiles (Figs. 3 and S1).

Based on these results, we developed an MLR model to identify a panel of metabolites that could discriminate between the two groups. The MLR model to discriminate CRC + P from HC showed significant differences in the predicted probabilities (Figs. 5 and S4, and Table S2). Because of the small sample size, especially in the HC1 and P groups, the results may have been caused by bias introduced during the random split to eliminate unexpected bias caused by the difference of the sample size. Therefore, we also developed an MLR model using the metabolites that were already selected in the protocol (Fig. 5A). We conducted 200 times two-fold CVs and confirmed the variance in the obtained AUC values (Figure S4AB). We performed resampling analysis and observed a larger variance in the AUC values compared to the two-fold CV (Figure S4C). In the two-fold CV analysis, all data were also randomly split into two datasets. While the ratio of HC, P, and CRC in the training and validation datasets were almost the same, these ratios in the resampling tests changed, which could have an impact on the accuracy of the resampling analysis. To address this, the model should be tested in a larger cohort with more participants in each of the groups.

The developed MLR model showed higher positive rates (87.1%) compared with the tumour markers CEA (69.9%) and CA19-9 (83.3%) (Table S3). The correlation coefficient (*R*) between CEA and CA19-9 was 0.408 (Table S4), which was the highest correlation coefficient between each tumour marker and the MLR model. Thus, the MLR model can be used as an independent factor to detect CRC patients.

Among the identified metabolites, amino acids were particularly elevated in CRC patients (Fig. 1A), especially in the advanced stages of cancer (Fig. 1B). Profiling of CRC tumour tissue revealed higher concentrations of amino acids than in the adjacent noncancerous tissues^37^. The intermediate metabolites of glycolysis and the TCA cycle are actively used for amino acid synthesis. The Warburg effect, commonly observed in tumour tissue, induces rapid synthesis of adenosine triphosphate (ATP) as an energy source, as well as of nucleic acids, amino acids, and lipids. Activated glycolysis has a rapid reaction rate of oxidative phosphorylation despite the low production efficiency per glucose, which is advantageous for the synthesis of these metabolites without the use of oxidative phosphorylation. The pathway analysis revealed that the TCA cycle exhibited a high difference between the HC1 and CRC groups and also between the CRC and P groups, which indicates that it is highly activated in CRC (Fig. 2). Among the TCA cycle metabolites, malate showed CRC-specific elevation (Fig. 4B). Citric acid, which was elevated in CRC (Fig. 4B), contributes to fatty acid production in cancer cells as an intermediate metabolite in the citric acid cycle. Cancer cells use fatty acids as an energy source, and they have increased de novo lipid metabolism. Many cancer cells undergo apoptosis or growth inhibition upon suppression of their lipid-metabolizing enzymes. The change in citrate concentration of serum samples from CRC patients was also reported^38^. Polyamines, such as *N*^1^-acetylspermidine and *N*^8^-acetylspermidine, were elevated in CRC (Fig. 1A), consistent with our previous study^9^. The synthesis of these metabolites was activated by the activation of ornithine decarboxylase and spermidine/spermine N1-acetyltransferase^18,39^ and also their elevation was frequently detected in urine samples collected from the patients with CRC^40^.

Amino acid synthesis by glutaminolysis, which is part of glutamine metabolism, plays an important role in cancer cell growth^41^. The high concentration of these metabolites in CRCs, which can metastasize to biofluids, especially in the advanced stages of CRC, is possibly caused by increased synthesis of metabolites. The concentration of kynurenine, a major degradation product of tryptophan, was significantly high in the urine collected from the patients with CRC (Fig. 4B). The synthesis of kynurenine is activated by MYC^15^ and this metabolite functions to promote cell proliferation in colon cancer cells^42^. Pantothenate, a type of vitamin B, was also significantly elevated in CRC (Fig. 4B). The elevated concentration of panthothenate in colon tissue was reported previously^43^. The elevation of urinary polyamines and amino acid-related metabolites observed in this study is consistent with the elevation in CRC tissue samples reported in various studies.

The MLR model included three metabolites (Table S1). Butyrate, which was found in the developed MLR model, is used as an energy source by colon cells in vivo. It is metabolized into acetyl CoA by β-oxidation and acts in the citric acid cycle to produce energy. Butyrate induces differentiation of undifferentiated T cells into regulatory T cells through epigenetic regulation^44^. Regulatory T cells function in the suppression of the immune response and play a role in autoimmune diseases, inflammatory diseases, and allergic diseases. Regulatory T cells may suppress the immune response to cancer cells and promote excessive metabolism in these cells.

The intermediate metabolite of leucine, 3-hydroxy-3-methylglutarate, was identified in the developed MLR model. Higher leucine levels were observed in the stools of patients with intramucosal cancer^45^. Carnosine, identified in the MLR model, suppresses cancer cell growth. Wu et al. reported that carnosine inhibits the activation of nuclear factor-kappa B (NF-κB) signalling, and subsequently inhibits metastatic cell adhesion and extravasation in CRCs^46^. Carnosine, which is involved in the suppression of metastasis, might also be useful as a CRC screening marker. Although these metabolites were identified in the present study as potential biomarkers for CRC, the biological mechanisms underlying their high concentration in the urine of CRC patients are not clear.

There are several limitations to this study. The samples size, especially the number of healthy controls and P patients, was small. In the present study, we did not compare the levels of these metabolites in other cancer types to establish the specificity of these biomarkers for CRC. Bias may also be introduced because of the demographic makeup of the cohort, and a larger number of subjects with various backgrounds should be compared to ascertain the specificity of the putative biomarkers. The prediction of metastasis and prognosis using biofluids is also challenging^47^, but was beyond the scope of the present study. The comparison of polyamines measured in the present study and in our previous study (Figure S5) reveals clear linearity between these data. *N*^1^-acetylspermidine, *N*^8^-acetylspermidine, and *N*^1^,*N*^8^-diacetylspermidine were successfully separated and quantified, whereas *N*^1^,*N*^12^-diacetylspermine was not successfully separated by CE under the present conditions.

In the present study, metabolites were identified in the urine of patients with CRC, P, and in HCs. The MLR was used to identify panels of metabolites with the ability to differentiate between these clinical groups. A method to differentiate between the different stages within the HC, CRC, and P groups remains elusive, and further analysis of a larger number of patients is required, particularly regarding early diagnosis at the polyp stage. Further research is expected to lead to the development of an accurate diagnostic method by measuring novel biomarkers for tumour identification. With the development of this future technology, it may be possible to confirm the presence of P or CRC in advance, before performing a colonoscopy, which would enable minimally invasive examination and treatment to be performed. This is expected to contribute to the further spread of medical examinations for CRC in Japan, and finally, reduce the harm caused by this disease.

## Supplementary information

Supplementary Information.
