# Supplementary material for: Urinary charged metabolite profiling of colorectal cancer using capillary electrophoresis-mass spectrometry
Source: Sci Rep. 2020 Dec 3;10:21057. doi: 10.1038/s41598-020-78038-2 (PMC7713069; doi:10.1038/s41598-020-78038-2)
Supplement: Supplementary file 1 — Supplementary Information. [file 41598_2020_78038_MOESM1_ESM.pdf]

Urinary charged metabolite profiling of colorectal cancer using capillary electrophoresis-mass spectrometry

Ryutaro Udo[1], Kenji Katsumata[1], Hiroshi Kuwabara[1], Masanobu Enomoto[1], Tetsuo Ishizaki[1], Makoto Sunamura[4], Yuichi Nagakawa[1], Ryoko Soya [1], Masahiro Sugimoto[2,3,*], Akihiko Tsuchida[1]

[1]Department of Gastrointestinal and Pediatric Surgery, Tokyo Medical University, 6-7-1, Nishijinjuku, Shinjuku, Tokyo, 160-0023, Japan

[2]Institute for Advanced Biosciences, Keio University, Tsuruoka, Yamagata 997-0811, Japan

[3]Research and Development Center for Minimally Invasive Therapies, Medical Research Institute, Tokyo Medical University, Japan

[4]Department of Gastroenterological Surgery and Transplantation Surgery, Tokyo Medical University Hachioji Medical Center, 1163 Tatemachi, Hachioji City, Tokyo, 193-0998, Japan

*__To whom correspondence should be addressed:__ Masahiro Sugimoto, PhD.
Research and Development Center for Minimally Invasive Therapies, Medical Research Institute, Tokyo Medical University, 6-1-1, Shinjuku, Tokyo, 160-0022, Japan,
Tel: +81-235-29-0528, Fax: +81-235-2

A)

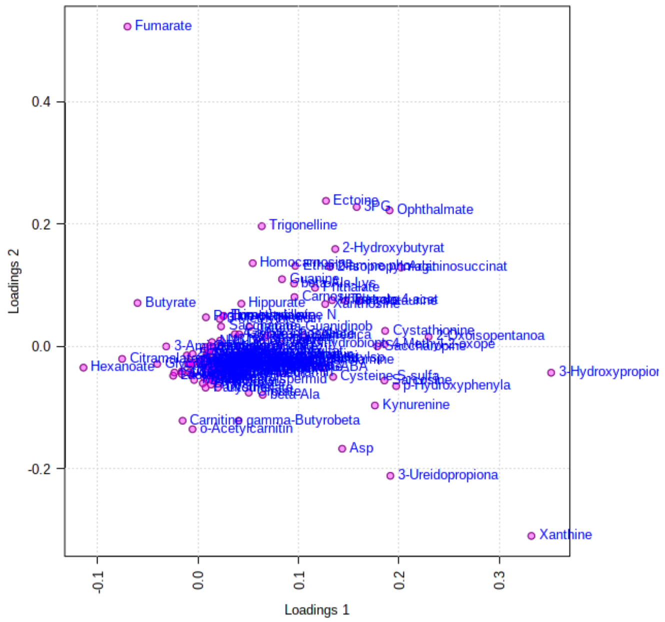

B)

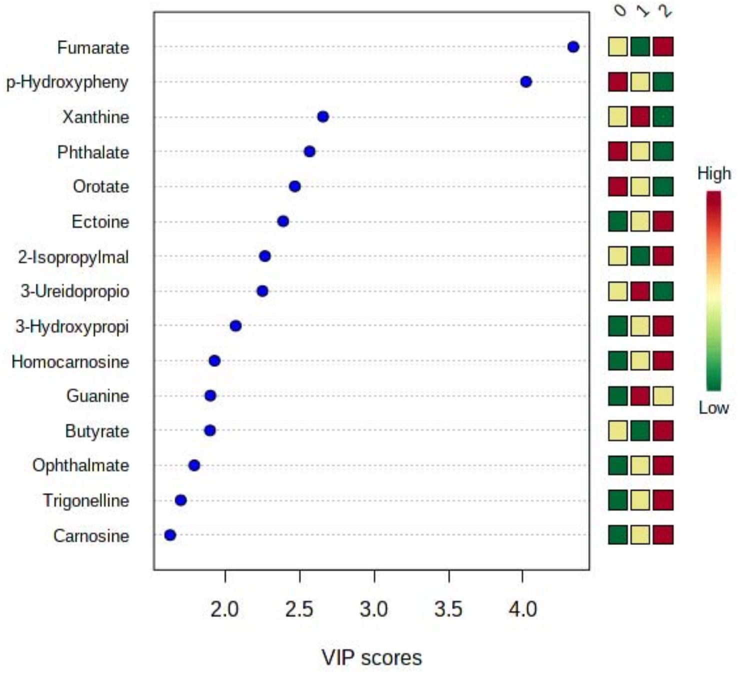

C)

Measure	1 comp	2 comps	3 comps	4 comps	5 comps
Accuracy	0.877	0.880	0.884	0.865	0.874
R^2	0.351	0.446	0.476	0.524	0.559
Q^2	0.236	0.287	0.283	0.269	0.217

Figure S1. Multivariable analysis. Loading plots of principal components analysis (PCA) **A)**, VIP score of partial least squares discrimination analysis (PLS-DA) **B)**, Relationship between components and PLS-DA **C)**. MetaboAnalyst (v.4.0, https://www.metaboanalyst.ca) was used.

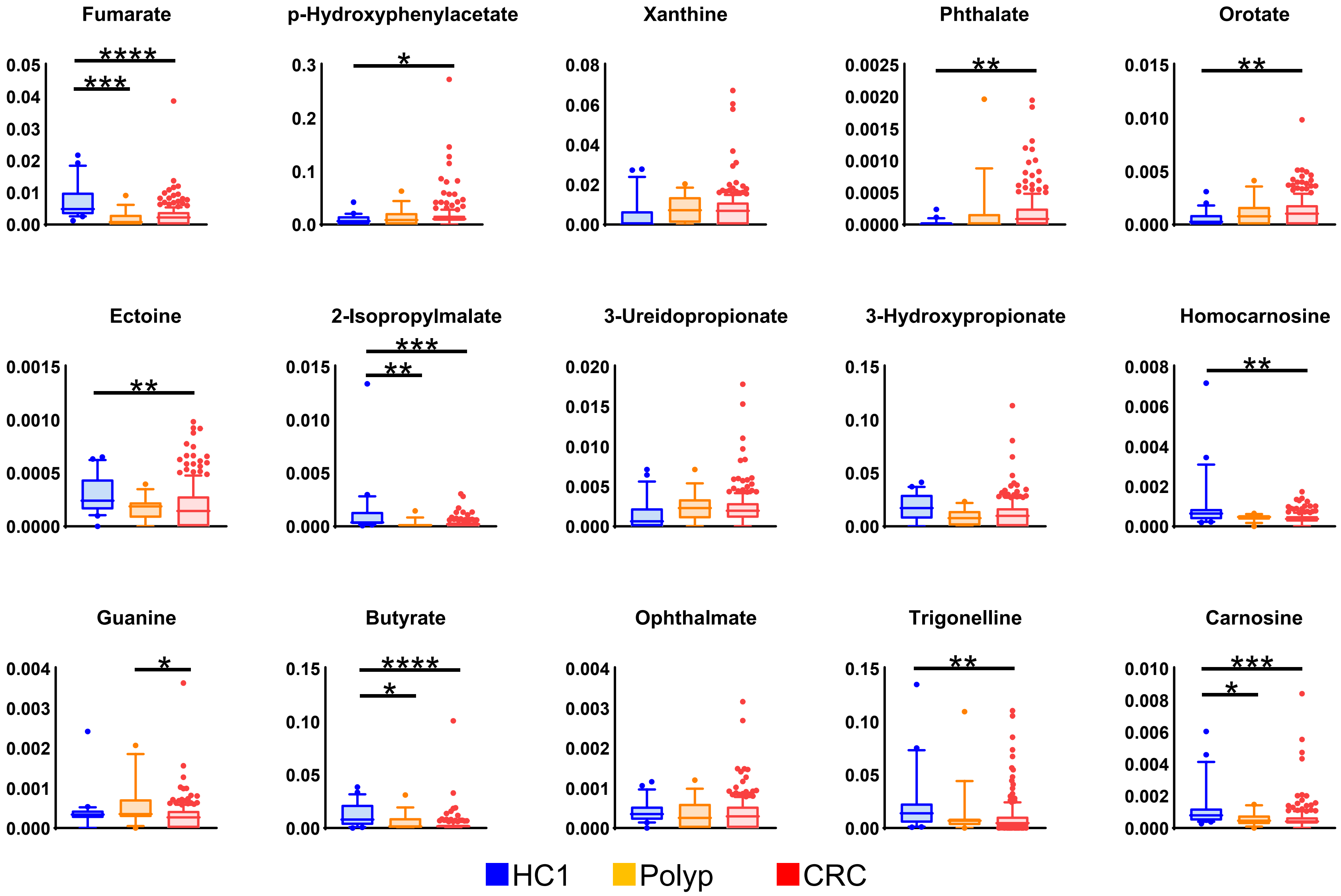

Figure S2 Top box plots of metabolite concentration within top 15 VIP scores in Figure S1. The comparison between healthy control (HC), polyp (P), and colorectal cancer (CRC) groups was performed using Kruskal–Wallis and Dunn's post-tests. ***, **, and * indicate *P* < 0.0001, *P* < 0.01, and *P* < 0.05 in the post-tests, respectively. GraphPad Prism (ver. 8.4.2, https://www.graphpad.com/) was used.

A)

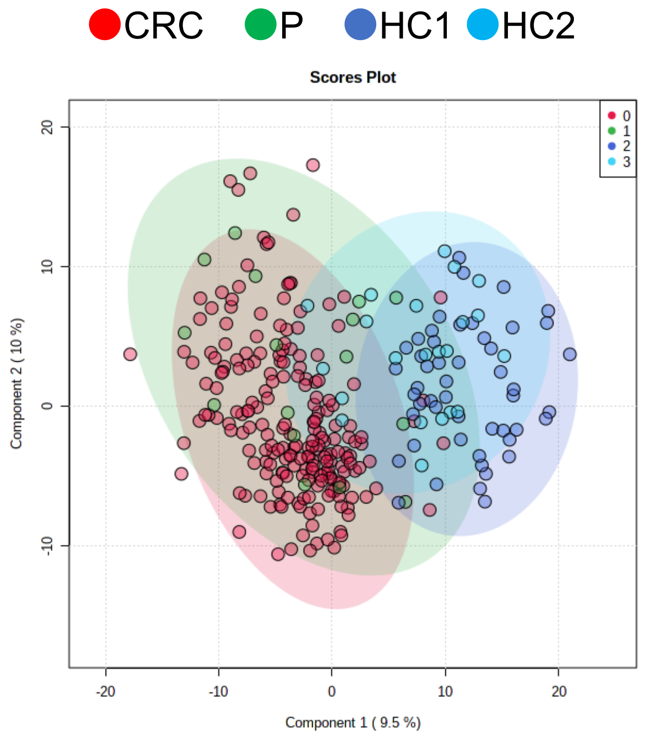

B)

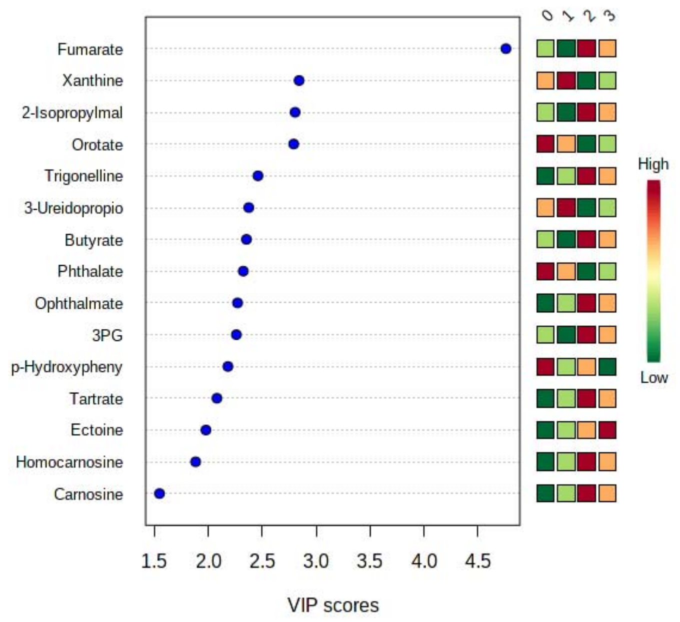

C)

Measure	1 comps	2 comps	3 comps	4 comps	5 comps
Accuracy	0.838	0.841	0.849	0.858	0.858
R^2	0.537	0.603	0.653	0.687	0.703
Q^2	0.500	0.5398	0.557	0.559	0.540

Figure S3. Partial least squares-discrimination analysis (PLS-DA) using all samples. Healthy control 2 (HC2) was added compared to the Figure 3. **A)** Score plots, **B)** VIP score, and **C)** R^2 and Q^2 values. MetaboAnalyst (v.4.0, https://www.metaboanalyst.ca) was used.

Figure S4

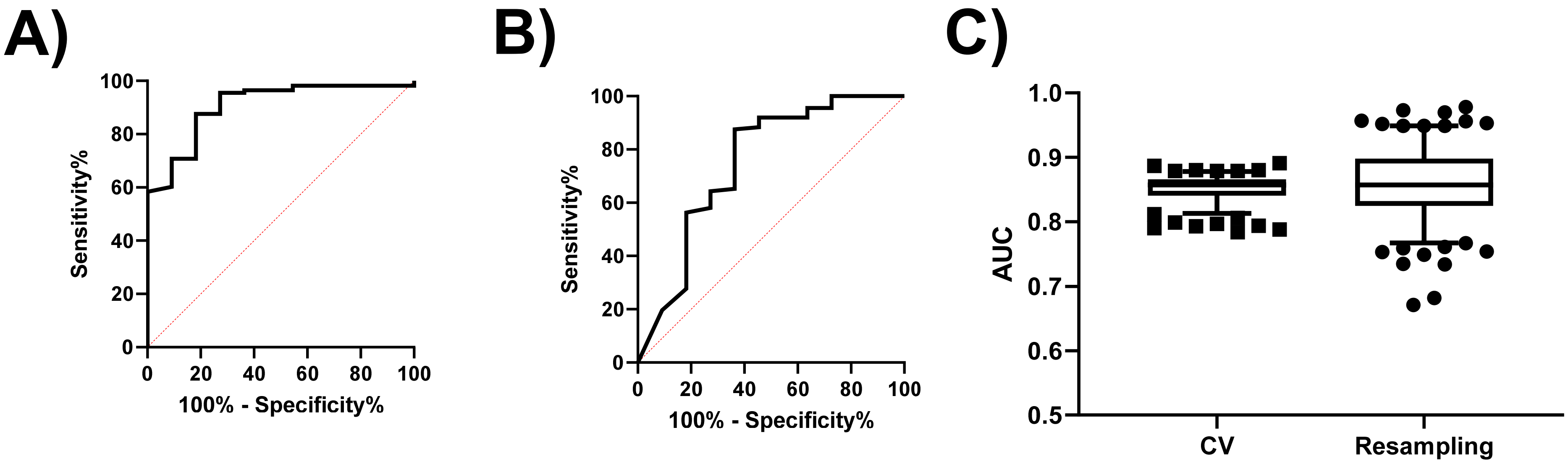

Figure S4. Receiver operating characteristic (ROC) curves of multiple logistic regression (MLR) models. **A**) 10-fold CV of training data and **B**) validation data. **C)** AUC values of two-fold cross validation and resampling test using whole dataset. Each test was conducted 200 times using random values. Horizontal bars of box plots indicate 95% CI, upper and lower quantile, and median of the data. The data comprising 95% of CI are depicted as dots. GraphPad Prism (ver. 8.4.2, https://www.graphpad.com/) was used.

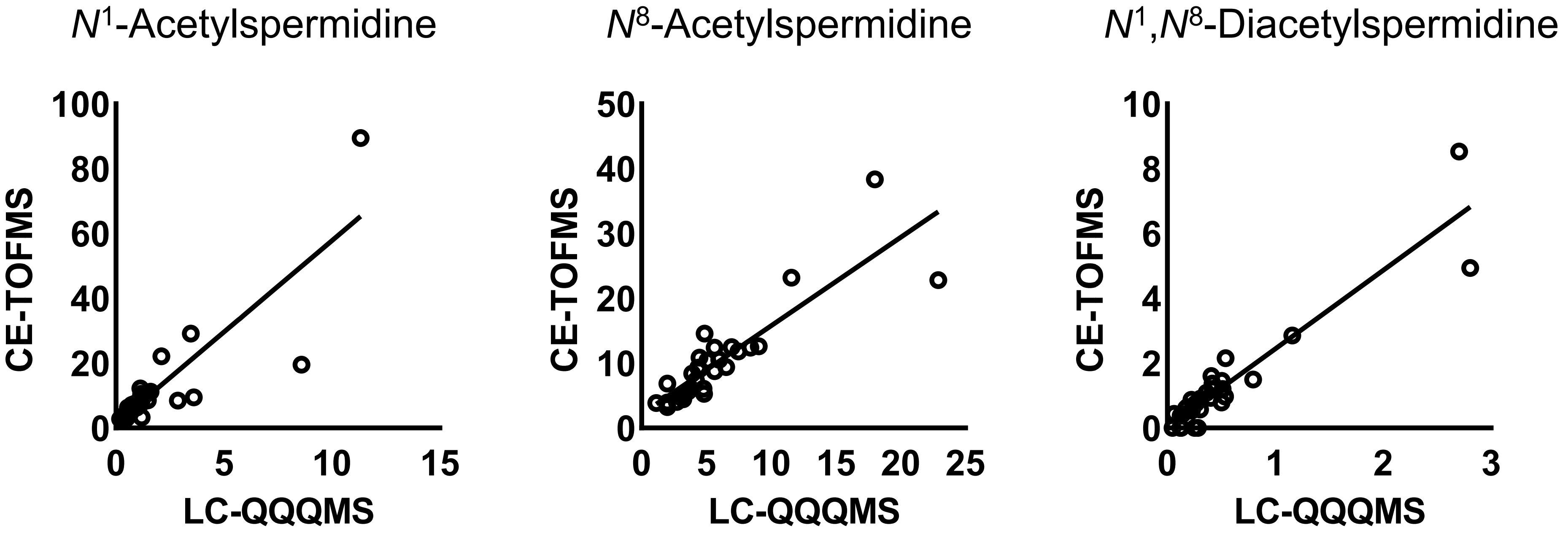

$Y = 5.639 \times X + 1.407$ $Y = 1.363 \times X + 2.145$ $Y = 2.424 \times X + 0.01534$

Figure S5. Comparison of urinary metabolite concentrations (μmol/g creatinine) using a randomly selected set of 30 samples. Data from the present study (Y-axis) and data from Nakajima et al. [Int J Mol Sci, 2018] (X-axis) are depicted. The equations below the graphs are linear regressions. GraphPad Prism (ver. 8.4.2, https://www.graphpad.com/) was used.

Table S1 Parameters of multiple logistic regression (MLR) model using training data and all data

	Parameter	95% CI		Odds radio	95% CI		P-value
**Training data**							
(Intercept)	-2.47	-4.83	-0.116	-	-	-	0.0397
Butyrate	-92.2	-1.48×10^{2}	-36.9	9.21×10^{-5}	3.48×10^{-7}	0.0244	1.10×10^{-3}
3-Hydroxy-3-methylglutarate	1.97×10^{3}	9.41×10^{2}	2.99×10^{3}	5.32×10^{10}	1.36×10^{5}	2.08×10^{16}	2.00×10^{-4}
Carnosine	-8.01×10^{2}	-1.49×10^{3}	-1.09×10^{2}	1.18×10^{-3}	3.48×10^{-6}	0.400	0.0233
**All data**							
(Intercept)	-0.663	-2.19	0.816	-	-	-	0.383
Butyrate	-1.00×10^{2}	-1.66×10^{2}	-47.3	4.03×10^{-5}	5.20×10^{-8}	8.47×10^{-3}	2.00×10^{-3}
3-Hydroxy-3-methylglutarate	1.14×10^{3}	6.63×10^{2}	1.71×10^{3}	1.73×10^{6}	4.16×10^{3}	2.04×10^{9}	<.0001
Carnosine	-4.69×10^{2}	-8.69×10^{2}	10.4	0.0193	6.66×10^{-4}	1.09	0.0283

Table S2 Prediction accuracy of multiple logistic regression (MLR) models.

	AUC	95% CI		*P*-value
Training	0.935	0.874	0.996	<0.0001
10-fold CV	0.907	0.825	0.988	<0.0001
Validation	0.748	0.563	0.933	6.80×10^{-3}

Table S3 Comparison of the positive rate between tumor markers and multiple logistic regression (MLR) model

	Positive (n)	(%)	Negative (n)	(%)	Total (n)
CEA	146	69.9	63	30.1	209
CA19-9	174	83.3	35	16.5	209
MLR	182	87.1	27	12.9	209

The thresholds for positive cases were 5 ng/mL for CEA, 37 U/mL for CA19-9, and 0.8766 (no unit) for MLR.

Table S4 Correlation coefficients between tumor markers, metabolites, and multiple logistic regression (MLR).

**CEA**	0.408	0.198	0.00790	0.124	0.188
	**CA19-9**	0.157	0.0532	0.0969	0.136
		**3-Hydroxy-3-methylglutarate**	-0.143	0.431	0.922
			**Carnosine**	-0.0109	-0.243
				**Butyrate**	0.256
					**MLR**

Spearman correlations (*R*) are shown.
